# Fishmeal Dietary Replacement Up to 50%: A Comparative Study of Two Insect Meals for Rainbow Trout (*Oncorhynchus mykiss*)

**DOI:** 10.3390/ani12020179

**Published:** 2022-01-12

**Authors:** Federico Melenchón, Eduardo de Mercado, Héctor J. Pula, Gabriel Cardenete, Fernando G. Barroso, Dmitri Fabrikov, Helena M. Lourenço, María-Fernanda Pessoa, Leidy Lagos, Pabodha Weththasinghe, Marcos Cortés, Cristina Tomás-Almenar

**Affiliations:** 1 Agro-Technological Institute of Castilla y León, Ctra. Arévalo s/n, 40196 Segovia, Spain; melramfe@itacyl.es (F.M.); eduardo.mercado@inia.es (E.d.M.); 2 Department of Zoology, Campus Fuentenueva, Facultad de Ciencias, University of Granada, 18071 Granada, Spain; pula@ugr.es (H.J.P.); gcardenete@ugr.es (G.C.); 3 Department of Biology and Geology, University of Almería, 04120 Almería, Spain; fbarroso@ual.es (F.G.B.); df091@ual.es (D.F.); 4 Division of Aquaculture, Valorisation and Bioprospection, Portuguese Institute for Sea and Atmosphere (IPMA, IP), Av. Dr. Alfredo Magalhães Ramalho 6, 1495-165 Algés, Portugal; helena@ipma.pt; 5 GeoBioTec, Departamento de Ciências da Terra, Faculdade de Ciências e Tecnologia, Nova School, 2829-516 Monte da Caparica, Portugal; mfgp@fct.unl.pt; 6 Department of Animal and Aquacultural Sciences, Faculty of Biosciences, Norwegian University of Life Sciences, P.O. Box 5003, NO-1432 Ås, Norway; leidy.lagos@nmbu.no (L.L.); pabodha.weththasinghe@nmbu.no (P.W.); 7 Laboratory of Immunology, Centre of Aquatic Biotechnology, Department of Biology, Faculty of Chemistry and Biology, University of Santiago of Chile, Av. Bernardo O’Higgins 3363, Santiago 9170002, Chile; marcos.cortes@usach.cl

**Keywords:** black soldier fly, mealworm, fishmeal replacement, rainbow trout, aquaculture, fish nutrition

## Abstract

**Simple Summary:**

The reduction of dependence on fishmeal as a main protein source for aquafeeds remains a big problem in reaching sustainable aquaculture. Several alternatives to this ingredient are being tested and developed, insects being one of the most promising. The present study included two different insect species (black soldier fly, *Hermetia illucens*, and yellow mealworm, *Tenebrio molitor*) in the formulation of diets for rainbow trout (*Oncorhynchus mykiss*) against one typical fishmeal-based diet. Different parameters related to both the efficiency of these diets and their physiological repercussions were analysed. Yellow mealworm proved to be the best alternative for the growth and nutrition of rainbow trout, possibly due to some changes described in protein utilization and intestine histology, while other parameters revealed the possible usage of insect meals as functional ingredients due to their repercussions on preventing tissue damage.

**Abstract:**

The demand of optimal protein for human consumption is growing. The Food and Agriculture Organization (FAO) has highlighted aquaculture as one of the most promising alternatives for this protein supply gap due to the high efficiency of fish growth. However, aquaculture has been facing its own sustainability problem, because its high demand for protein has been traditionally satisfied with the use of fishmeal (FM) as the main source. Some of the most promising and sustainable protein substitutes for FM come from insects. The present manuscript provides insight into an experiment carried out on rainbow trout (*Oncorhynchus mykiss*) with a 50% replacement of FM with different larvae insect meals: *Hermetia illucens* (HI), and *Tenebrio molitor* (TM). TM showed better results for growth, protein utilization and more active digestive function, supported by intestinal histological changes. Liver histology and intermediary metabolism did not show relevant changes between insect meals, while other parameters such as antioxidant enzyme activities and tissue damage indicators showed the potential of insect meals as functional ingredients.

## 1. Introduction

Albeit at a slower speed than some decades ago, the global population is expected to keep increasing and reach 8.5 billion in 2030, 9.7 billion in 2050, and 10.9 billion in 2100 [1]. As a consequence of this increment, the demand of adequate protein for human consumption is also increasing. Aquaculture is one of the most promising alternatives to satisfy this demand due to the high efficiency of fish growth [2], the rapid development of the aquaculture industry itself, and the adequate calories—protein ratio of fish [3]. However, because many of the fish cultivated for human consumption require high protein levels to grow appropriately, aquaculture has been facing its own sustainability problems in the last few decades. These protein requirements have been traditionally satisfied with the use of fishmeal (FM) from wild-caught fish and as a by-product of extractive fishing practices [4]. Due to the fast growth of aquaculture, these ingredients are considered as non-sustainable in the long term.

Many efforts have been carried out from both research and aquaculture industries to partially replace FM with sustainable ingredients in fish feeds, without impairing fish growth and while giving insight into these sustainable ingredients. Alternatives such as vegetable ingredients [5,6], yeast [7,8], or microalgae [9,10] are some of the ingredients that are being studied currently. Following this line, the present study is focused on insects as one of the most interesting protein substitutes for FM [11,12,13,14]. Setting aside the interspecific differences, as well as the harvesting time of larvae [15], the amino acidic proportions of the most typically studied insects tend to match that of FM [16,17]. Insects also reproduce and grow easily, have very efficient growth ratios, and require low amounts of space and energy to be produced [18]. Hence, their potential as a good source of sustainable animal protein is promising. Because the Food and Agriculture Organization (FAO) has mentioned zero hunger, sustainable communities, and life below water as three of its 17 Sustainable Development Goals of the 2030 Agenda [19], it is easy to assume that both aquaculture and insect production might consequentially play important roles in the upcoming years or decades.

Several manuscripts have proven the efficiency of insect meals (IMs) in different fish species [20,21,22,23,24], revealing the importance of both the insect and the fish species involved. For salmonids, the inclusion of IMs in feeds is, in general, well accepted. As an example of unaffected growth, Terova [25] replaced up to 30% FM with *Hermetia illucens* (HI) in rainbow trout feed (*Oncorhynchus mykiss*). In the case of Atlantic salmon (*Salmo salar*), it was already proven that FM could be replaced entirely, using HI as one of the chosen protein sources [26]. Another experience [27] tested two hydrolysed IMs (yellow mealworm, *Tenebrio molitor* (TM) and superworm, *Zophobas morio*) on fingerlings of sea trout (*Salmo trutta* m. *trutta*) at 40% FM replacement and noticed almost no changes in growth or protein use. Moreover, another experiment [28] did not note changes in growth for rainbow trout, substituting FM completely with TM. These and other published data support the idea that, with the due differences among species, a partial replacement of FM with IM has no adverse effects on the growth of most fish. Moreover, functional properties such as a possible enhancement of both the immunological and the antioxidant systems have been attributed to IMs, possibly due to compounds such as chitin or its derivatives [14,21,29,30,31,32,33].

The European Commission approved the use of seven insects as ingredients in aquafeeds [34]. Due to their relative availability, HI and TM are two of the most broadly studied insects for animal nutrition. Thus, the IM industry has a big potential and requires research studies to validate the use of IMs as an alternative ingredient in feed for aquaculture.

Following the results of a previous study with 15–30% FM replacement (5–10% IM inclusion level) [32], but increasing the FM replacement to 50% (18% IM inclusion level) in feed for rainbow trout, the present manuscript provides insights on the effects of two different IMs for several aspects, from growth to final composition of the fillets, while evaluating the physiological status of the fish and their possible consequences on health and welfare status.

## 2. Materials and Methods

### 2.1. Experimental Diets

Whole dried insects from two different species in larval stage, *Hermetia illucens* (HI; Entomotech S.L., Almería, Spain) and *Tenebrio molitor* (TM; Mealfood Europe S.L., Salamanca, Spain) were used for this study, processed as insect meals (IMs). IMs were analysed before the formulation of the diets (Table 1). A total of three isoproteic (43.3%) and isolipidic (17.4%) diets were formulated (Table 2): a control diet with no IM (C), and two diets with 18% diet inclusion (50% fishmeal replacement) of the cited IMs: H18 (HI), and T18 (TM). Ingredients were provided by ‘Lorca Nutrición Animal S.A.’ (Murcia, Spain). Methionine and lysine were added to diets to meet the nutritional requirements of rainbow trout [35,36], manufactured by LifeBIOENCAPSULATION S.L. (Almería, Spain), and extruded as pellets of 3 mm. The dough was passed through a single screw laboratory extruder (Miltenz 51SP, JSConwell Ltd., Palmerston North, New Zealand). The extruder barrel had four sections, with a temperature per section of (from inlet to outlet) 100 °C, 95 °C, 90 °C and 85 °C, respectively. Pellets were kept in a drying chamber at 30 °C for 24 h (Airfrio, Almería, Spain) and stored in sealed plastic bags at −20 °C until they were used.

### 2.2. Experimental Animals and Rearing Conditions

A total of 360 female rainbow trout with an initial weight of 14.6 ± 0.2 g from a commercial farm (Piscifactoría Fuente del Campillo, Guadalajara, Spain) were transported to the experimental facilities of the Aquaculture Research Centre of “Instituto Tecnológico Agrario de Castilla y León” (ITACyL). Fish stayed in acclimation for 15 days before the beginning of the growth trial, and then they were randomly allocated into 12 cylindrical fiberglass tanks (four replicas per treatment; 500 L) of a recirculating system, in groups of 30 animals. Once a day (9 a.m.), fish were fed by hand until apparent satiation was reached (maximum of 3% daily feed intake). During the growth trial (77 days), water temperature (12.5 ± 1 °C), water dissolved oxygen (9.2 ± 1 mg/L), and room photoperiod (12 h light: 12 h dark) were monitored. Water ammonia and nitrite levels were analysed daily, and kept at optimal levels (ammonia < 0.1 mg/L and nitrite < 0.1 mg/L). The care and handling of rainbow trout were conducted according to specific regulations: The Directive of the European Union Council (2010/63/EU) [37] and the Spanish Government (Real Decreto 53/2013) [38]. The experiment was approved previously by the Bioethical Committee of “ITACyL” (Authorization number: 2017/19/CEEA).

### 2.3. Growth Trial and Samples Collection

Mortality and feed intake were monitored on a daily basis. Fish were measured and weighed every 21 days through a simple biometry procedure with a graduated ictiometer (±0.1 mm) and scale (±0.1 g), being previously fasted for one day and anesthetized with tricaine methanesulfonate (MS-222; 180 mg/mL). In order to take samples of the different tissues, the fish were sacrificed by an overdose of MS-222 (300 mg/mL).

Before the feeding trial, eight fish were randomly sacrificed to analyse the initial value of the protein in the fillet.

During the final two weeks of the experiment, faeces were gathered every 24 h in a settling column using a modified Guelph method [39], and frozen at −80 °C until they were analysed. At the end of the experiment, eight fish per diet (2 fish per tank) were randomly sampled and sacrificed. According to time sequence, the following were collected to be analysed individually: skin mucus, blood, liver, stomach, intestine with pyloric caeca, and fillet samples. Skin mucus samples were collected by scraping the dorso-lateral surface of the fish skin from cranial to caudal according to de Mercado et al. [40] and frozen at −80 °C until processing. Blood samples were collected with heparinized syringes and their plasma was separated by centrifugation at 3500× *g* and 4 °C, for 15 min. Individual plasma samples were frozen at −80 °C until their analysis.

For enzyme determinations, samples were frozen in liquid nitrogen and kept at −80 °C until they were analysed. For tumour necrosis factor-alpha determination (TNF-α), distal intestine samples were kept in Allprotect Tissue Reagent (QiaGEn) and stored at −20 °C until protein extraction. The samples for histomorphology analyses were fixed in 4% buffered formalin for 48 h before dehydration and processing. For chemical analyses, the samples were directly frozen at −80 °C.

### 2.4. Histomorphology

#### 2.4.1. Samples Processing

The fixed samples were dehydrated in increasing ethanol solutions (25%, 50%, 75%, and 100%) and embedded in synthetic paraffin. Histological sections (3–4 µm) were obtained by a rotary microtome (FINESSE ME+ Thermo Scientific©, Waltham, MA, USA), stained by hematoxylin and eosin technique for histomorphology studies and observed with light microscopy. All of the evaluations were performed by graded objective lens in five random regions for each stained tissue section with an Olympus EP50 microscope camera and an Olympus CX31 microscope.

#### 2.4.2. Distal Intestine and Pyloric Caeca Histomorphology Analyses

Quantitative studies included the measurement of heights of villi and enterocytes, as well as widths of villi, *stratum compactum*, muscular layers (longitudinal and circular), and lamina propria as mean of three measures (apical, intermediate, and basal). The level of inflammatory infiltration in lamina propria, the level of loss of supranuclear vacuolization of enterocytes, and the relative position of enterocyte nuclei were measured through a subjective analysis.

#### 2.4.3. Liver Histomorphology Analysis

Hepatocyte cytoplasm and hepatocyte nucleus measures were taken as quantitative variables. A qualitative analysis concerning the search of inflammatory patterns (necrosis and inflammation focuses) and hepatocyte intranuclear vacuolization was also carried out.

### 2.5. Analytical Determinations

For intermediary metabolism and antioxidant status, liver samples were individually homogenized in nine volumes of ice-cold 100 mM Tris-HCl buffer, containing 0.1 mM EDTA and 1 g/kg (v/v) Triton X-100, pH 7.8. This was followed by centrifugation at 30,000× *g* for 30 min, at 4 °C. For further enzyme assays, the supernatants were stored at −80 °C as aliquots.

The concentration of soluble protein in samples was determined by Bradford method [41], employing bovine serum albumin as a standard.

#### 2.5.1. Chemical Analyses

AOAC methods [42] were used to analyse fat content and moisture of IMs, diets, and fish fillets. Protein content was determined with the Dumas method [43], using a nitrogen analyser (FP 528, LECO, St. Joseph, MO, USA), and with a conversion factor of 4.67 for HI, 4.75 for TM [44], and 6.25 for feeds and faeces. Acid-insoluble ash was used as marker in feeds and faeces to determine the apparent digestibility of the protein [45]. Phosphorus (P) was determined by molecular absorption spectrophotometry according to ISO standard [46], with a spectrophotometer (UV/Vis UV2, UNICAM, Cambridge, UK). Calcium was determined as described by Pessoa [47], with X-ray fluorescence method of Dispersive Energy. The method described by Gamage and Shahidi [48] was used to isolate chitin from IM, which was washed with acetone, dried, and weighed afterwards. For amino acids, samples of HI and TM were hydrolysed with 6 N HCl for 22 h at 110 °C [16]. The determination was performed by ion-exchange liquid chromatography and postcolumn continuous reaction with ninhydrin (Biochrom 30; Cambridge, UK). Tryptophan was not determined.

#### 2.5.2. Digestive Enzymes Determination

Intestine with pyloric caeca and stomach were processed separately to determine digestive enzymes. Samples were first individually homogenized at 4 °C with distilled water (250 mg/mL). Acid protease activity was determined from stomach extracts, while amylase and alkaline protease activities were determined from the intestine and pyloric caeca extracts. The activity of amylase was determined through the Somogy–Nelson method [49], with soluble starch 20 g/kg as substrate, defining one unit of activity as the quantity of enzyme able to produce 1 μg of maltose per minute and mg of protein. Walter method [50] was used to measure the activity of alkaline protease, employing casein 10 g/kg as substrate. Anson method [51] was used to measure the activity of acid protease activity, with hemoglobin 5 g/kg as substrate. For both proteases, one unit of activity was defined as 1 μg of tyrosine produced per minute and mg of protein. The standard temperature for all digestive enzyme analyses was 37 °C.

#### 2.5.3. Liver Intermediary Metabolism

The method described by Furné [52] was used to determine the enzymatic activity of fructose 1,6-bisphosphatase (FBPase; EC 3.1.3.11), pyruvate kinase (PK, EC 2.7.1.40), glutamate pyruvate transaminase (GPT; EC 2.6.1.2), glutamate oxaloacetate transaminase (GOT; EC 2.6.1.1), and glutamate dehydrogenase (GDH; EC 1.4.1.2). Enzymes were analysed at 25 °C, and changes in absorbance were monitored with a PowerWaveX microplate scanning spectrophotometer (BioTek Instruments, Winooski, VT, USA) to determine the enzyme activity.

#### 2.5.4. Non-Specific Immune Status

##### Plasma Immunological Determinations

Lysozyme activity was performed using a turbidometric method [53] with *Micrococcus lysodeikticus* (Sigma, St. Louis, MO, USA). After reaction for 20 min at 35 °C, the absorbance was measured at 450 nm. A standard curve with hen egg-white lysozyme was used.

Total esterase activity was assayed according to Mashiter and Morgan [54] at 25 °C. P-nitrophenyl acetate (0.8 mM) was used as a substrate, and 1.6 mM acetazolamide as an inhibitor of carbonic anhydrase activity. The absorbance increase was measured at 405 nm for 5 min, after incubation for 10 min.

Anti-protease was measured according to Thompson [55]. The production of 4-nitroaniline was determined by the variation of the OD (optical density) at 410 nm for 30 min. Trypsin activity in absence of plasma was used as control (CAS 90002–07–7, Acofarma, Spain).

Phosphatase activity was determined according to Huang [56]. P-nitrophenyl phosphate (Sigma) was used as a substrate, a buffer at pH 10 (NaHCO_3_/NaOH 0.05 M, MgCl_2_ 1 mM) was used for alkaline phosphatase activity, and a buffer at pH 5 (CH_3_COOH/CH_3_COOHNa 0.1 M, MgCl_2_ 1 mM) to measure acid phosphatase activity. The measurement was performed at 405 nm for 30 min, at 37 °C.

Peroxidase activity was determined according to Mohanty and Sahoo [57]. A solution of 20 mM TMB (3, 3′, 5, 5′-Tetramethylbenzidine) was used as substrate. The samples were read at 450 nm after blocking reaction for 2 min. Plasma-free standard samples were measured as controls. The activity was expressed in OD (optical density).

Total immunoglobulin was determined according to Panigrahi [58]. After precipitation with polyethylene glycol, the immunoglobulins were separated from the total proteins. Total immunoglobulin content was calculated by subtracting the protein content resulting from the total protein content in the untreated plasma.

##### TNF-α Detection in Distal Intestine and Skin Mucus

For protein extraction, samples of the distal intestine and skin mucus were homogenized using beads and an ice-cold lysis buffer (Tris 20 mM, NaCL 100 mM, Triton X-100 0.05%, EDTA 5 mM, protease inhibitor cocktail 1X), in a bead mill homogenizer (Qiagen RETSCH tissuelyser). The homogenate was centrifuged for 25 min, at 12,000× *g* and 4 °C. The soluble proteins contained in the supernatant were stored at −20 °C until use. The cytokine TNF-α was determined following the indirect ELISA method described by Morales-Lange [59], with slight modifications according to Weththasinghe [33]. Briefly, 100 µL of sample diluted to 45 ng/µL in a carbonate buffer (60 mM NaHCO_3_, pH 9.6) were seeded into 96-well plates (NUNC MAXISORPTM, Invitrogen), and incubated overnight at 4 °C. After blocking (5% Blotting-Grade Block, BioRad, Hercules, CA, USA; 2 h at 37 °C), plates were incubated for 90 min at 37 °C with 50 μL of the primary antibody (rabbit anti-TNFα, diluted 1: 200). Next, 50 μL of the secondary antibody (mouse anti-rabbit IgG-HRP, diluted 1: 7000) were added and incubated for 60 min at 37 °C. Finally, 100 μL of chromagen substrate 3,3′,5,5′- tetramethylbenzidine single solution (TMB, Thermofisher, Waltham, MA, USA) was added and incubated for 30 min at room temperature. The reaction was stopped with 50 mL of 1 N sulfuric acid and read at 450 nm on a Spectra Max microplate reader (Spectra Max M2; Molecular Devices, San José, CA, USA). The calibration curve was performed using serial dilutions of the corresponding epitope peptide ranging from 0 µg/mL to 1.2 µg/mL.

#### 2.5.5. Liver Antioxidant Status and Fish Welfare Indicators

The procedure described by Pérez-Jiménez [60] was followed to determine superoxide dismutase (SOD, EC 1.15.1.1), catalase (CAT, EC 1.11.1.6), glutathione peroxidase (GPX, EC 1.11.1.9), glutathione reductase (GR, EC 1.6.4.2), and glucose-6-phosphate dehydrogenase (G6PDH; EC 1.1.1.49). The enzyme analyses were carried out at 25 °C, and the enzyme activity was determined using a PowerWaveX microplate scanning spectrophotometer (BioTek Instruments, Winooski, VT, USA), through the monitorization of absorbance changes. Preliminary assays allowed the establishment of the optimal substrate and protein concentrations to measure the maximal activity of each enzyme. The millimolar extinction coefficients used for NADH/NADPH, DTNB, and H_2_O_2_ were 6.22, 13.6, and 0.039/mM·cm, respectively. The enzyme needed to inhibit half of the ferricytochrome C reduction rate was defined as one unit of SOD activity. For other enzymes, the amount of enzyme required to transform 1 μmol of substrate per minute was defined as one unit of enzyme activity. Malondialdehyde (MDA) level was used to quantify lipid peroxidation. MDA reacts in the presence of thiobarbituric acid to produce coloured thiobarbituric acid reacting substances (TBARS).

Plasma glucose and lactate levels were analysed with commercial colorimetric kits, following the instructions of the manufacturer (Glucose-TR, ref. 41011, Spinreact; Lactate, ref. 1001330, Spinreact). The absorbance was measured using a microplate reader (ELx800TM; BioTek Instruments, Inc., Winooski, VT, USA), in 96-well microplates.

### 2.6. Statistical Analysis

The software used for the statistical analyses was SAS system version 9.0 (SAS Institute Inc., Cary, NC, USA). A general linear model (PROC GLM) analysis of variance (ANOVA) was used to process the data, and the comparison of the means was performed by a Tukey test. Differences were considered significant when the *p*-value was <0.05. Values are showed as mean ± standard error of the mean.

## 3. Results and Discussion

### 3.1. Growth Performance

In general, the performance of all diets was within normal values, with an efficient FCR (Table 3). Fish fed with T18 showed the best overall growth performance with very similar values to C while H18 showed lower numbers for growth, being statistically different from T18, or even to C when talking about SGR, FCR, and the apparent digestibility coefficient of the protein (ADC_prot_). Even though there are small discrepancies in the literature about the performance of HI as an ingredient for fish [61,62,63,64], the present results seem to follow the general conclusions of other trials in rainbow trout. Rainbow trout seem to have a higher tolerance to the inclusion of TM in diets [65,66] than that of HI [61,67], which could be due to the different levels of chitin in the composition of the insects, or their amino acid profiles. Chitin might have a positive influence over fish physiology as a functional ingredient [68], but the presence of this molecule tends to lower the digestibility of crude protein [69,70]. Because HI has higher levels of chitin in its body composition than TM (Table 1), this, together with results in other experiences [61,65,67,71] suggest that a 15–18% inclusion of HI in rainbow trout feed is a possible maximum level of inclusion for this species, while an 18% of TM or even more, is still compatible with optimal growth performance. In addition, the digestibility of the protein was higher in TM than HI; although the amino acid profile between IMs differed, the diets were supplemented with methionine and lysine to cover the nutritional requirements. This should had led to a similar growth between insect-based treatments, but the higher growth of T18 over H18 means that its higher digestibility played an important role, and consequently led to a higher growth than HI.

The mortality during the trial was around 3% without remarkable differences between diets (data not shown).

### 3.2. Histomorphology

#### 3.2.1. Distal Intestine and Pyloric Caeca

**Figure 1:**
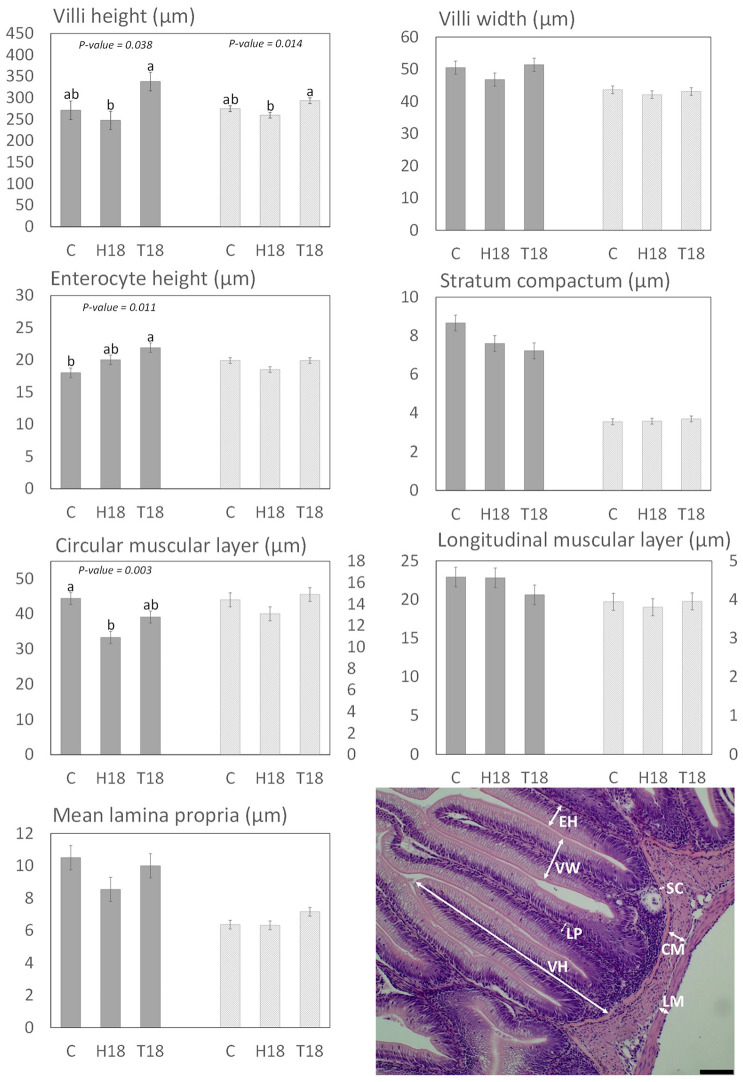
Histomorphology of rainbow trout gut. C: control diet; H18: 18 % HI inclusion; T18: 18 % TM inclusion. Grey bars: distal intestine measures; striped bars: pyloric caeca measures. a, b Show statistically significant differences among diets (*p* < 0.05); Values expressed as mean ± standard error of the mean (SEM; *n* = 4 tanks per diet, 2 fish per tank). Microphotograph representative of measures for gut: villi height (VH), villi width (VW), enterocyte height (EH), *stratum compactum* (SC), circular muscular layer width (CM), longitudinal muscular layer width (LM), lamina propria width (LP). Scale bar = 100 µm.

**Figure 2:**
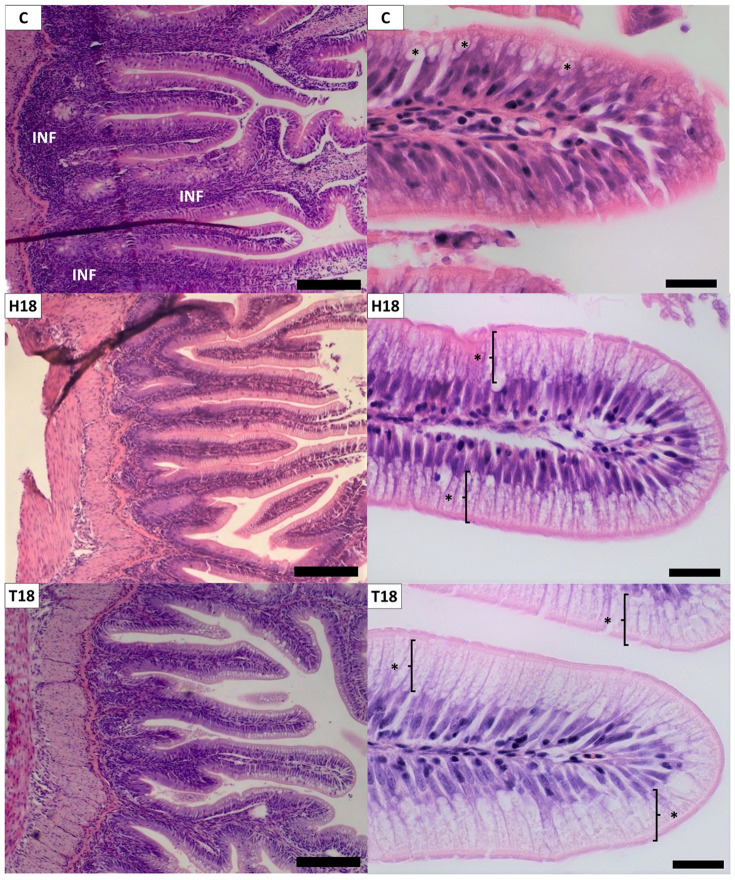
Histomorphology of rainbow trout distal intestine. C: control diet (no IM inclusion); H18: 18 % HI inclusion; T18: 18 % TM inclusion. Microphotographs of qualitative analyses: left photographs represent a comparative view of inflammatory infiltration (INF), pictures taken at 40× magnification, scale bars = 100 µm; right photographs represent a comparative view of vacuoles (marked with asterisks) in enterocytes, pictures taken at 400× magnification. Scale bars = 10 µm.

On distal intestine (Figure 1), no significant differences were described for villi, *stratum compactum*, longitudinal muscular layer, or lamina propria widths. Villi height was higher for T18 fish than H18 (*p* < 0.05), with an intermediate value for C fish. Enterocyte height was higher in T18 than in C (*p* < 0.05), with no significant differences between H18 and T18. The circular muscular layer was wider on C than on H18, while the total muscular layer was wider on C than on both IM treatments (*p* < 0.05). For qualitative analyses (Figure 2), slightly higher levels of both inflammatory infiltration and loss of intracellular vacuolization were highlighted for C diet. The different degrees of supranuclear vacuolization did not affect the enterocyte structure because most nuclei were positioned on the basal part of the enterocytes, showing no differences between treatments.

Few significant differences were found in pyloric caeca compared to the distal intestine. T18 showed the highest values for villi height (compared to H18; *p* < 0.05), while the rest of the results remained stable (Figure 1). For the qualitative analyses, no differences were observed for inflammatory infiltration or intracellular vacuolization, and most nuclei were described on the intermediate part with no differences between treatments.

The histology of insect-fed fish intestine has been extensively studied in the last few years [27,33,63,72,73,74]. However, due to the large number of variables involved in the studies, such as the fish species, the insect used to elaborate the feeds, the chosen intestine sections, or the analysed parameters, there is still work to be conducted. Villi height is one of the most frequently analysed parameters, being an indicator usually associated with gut health and growth performance. In this way, the results of this study match partially those of the current literature, because it has been described that similar inclusions of HI in fish feed tend to decrease villi height [67,71,75,76,77]. There are other cases in which no changes or even an increase in villi height was described with the addition of HI meal [63,78,79]. The case of TM is less studied and it seems that, with one exception [63], most studies describe no changes in villi height when dealing with this ingredient [27,73,80].

On pyloric caeca, the results were similar. However, this is a less studied variable for insect-fed fish, and to our knowledge, only two studies evaluated this parameter after a growth trial with HI treatments; one that matched the data of this study [77], and one that offered opposite results [81]. Considering that digestive efficiency and growth are directly related to gut anatomy alterations such as a decrease in the absorption surface [82,83,84], it is no surprise that the results of the present manuscript agree with the lower growth performance and protein digestibility described for HI, as well as the higher results on TM.

The circular muscular layer was also affected in the present study. The main finding was a decrease in the width for H18 with significant difference with respect to C diet (*p* < 0.05). Because contraction and relaxation of circular and longitudinal muscular layers lead to peristaltic movements, a different width of the circular muscular layer could alter the movement of the feed along the gut, affecting the intestinal bacterial growth [85] and ultimately the digestibility of the nutrients, consistent with the lower digestibility of the protein observed in fish fed with H18. Similar results were showed by Lu [76], while other authors have not described changes in muscular layer width [27,79,81]; there is even a case in which different insect species gave different results [63] with the inclusion of IMs. This divergence of data is probably caused by the different species of fish used.

It is interesting to notice that the differences in the degree of enterocyte supranuclear vacuoles loss (fewer vacuoles in C) are in consonance with the results on enterocyte height (lower in C, with a significant difference between C and T18). The presence or absence of lipidic vacuoles in enterocytes has been related to their height for other ectothermic species [86,87]. In this way, the different nature of the fat between diets (insect fat in H18 and T18), and their absorption process may have played an important role in the degree of supranuclear lipidic vacuoles in enterocytes. Furthermore, the work of Kumar [88] described how IMs could cause a protective effect against the problems of soybean meal [89] in salmonids. Considering that C diet also showed a slightly higher submucosa inflammatory infiltration, the lower degree of vacuolization and the consequential lower enterocyte height with respect to IM diets could be due to the lack of this protective effect. The three diets had a relatively high amount of vegetable ingredients, but even though H18 and T18 had the highest amounts of these ingredients, they showed the lowest levels of inflammatory signs.

#### 3.2.2. Liver

**Figure 3:**
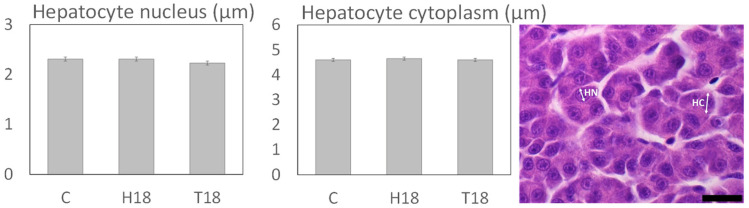
Histomorphology of rainbow trout liver. C: control diet (no IM inclusion); H18: 18 % HI inclusion; T18: 18 % TM inclusion. Values are expressed as mean ± standard error of the mean (SEM; *n* = 4 tanks per diet, 2 fish per tank). Microphotograph representative of measures for liver: hepatocyte nucleus (HN) and hepatocyte cytoplasm (HC) diameters. Scale bar = 10 µm.

No significant differences were found in any of the measured variables: hepatocyte nucleus and cytoplasm diameters, inflammatory patterns, or level of hepatocyte vacuolization (Figure 3). Liver histology has also been extensively studied for insect-fed fish [71,72,74,75,81]. One of the most frequent findings in several fish species, including rainbow trout, is that an increasing proportion of IMs in the feed tends to increase the number of lipidic vacuoles in hepatocytes, while other related variables are mostly unaffected [71,78,81]. This discrepancy with our results could be due to the different size and feeding period of the fish involved, because the cited study in rainbow trout was performed with bigger fish. The work of Kumar [88], however, had similar conclusions to those of the present study.

### 3.3. Digestive Enzymes

Acidic proteases showed no significant differences between treatments (Table 4; *p* > 0.05), while alkaline proteases and amylase showed significant increases in T18 treatment. The higher values of alkaline proteases for T18 are in consonance with the bigger digestibility of the protein demonstrated by this diet over H18 (Table 1). Because IM diets showed higher values of alkaline proteases than C, as well as lower acidic–alkaline ratios (non-statistically significant for H18), this supports the idea that insect-based diets suffered a more active digestion in the intestine. Contrarily, the work of Coutinho [23] showed a lower activity of total alkaline protease, trypsin, and lipase even on low inclusions of TM (defatted) for meagre (*Argyrosomus regius*). However, this work and the work of Guerreiro [22] showed that meagre might not be the best candidate to use IMs as a source of protein, which supports the point that differences between species must always be considered. In the present study, the increase in alkaline proteases could have been caused by the added effort of having to break the β(1–4) glycosidic bonds of chitin polymers, and especially its metabolites, because it was proven that chitinase activity is low in rainbow trout [90]. However, because chitinase (mostly located in the stomach) and chitobiase (mostly located in the intestine) activities were not measured, this would remain as a theory to encourage more future research.

The results for amylase activity follows closely the tendency of a previous study [32]. It has been described that amylase activity increases with the amount of carbohydrates in the diet [91]. Moreover, Rapatsa and Moyo already proved [92,93] that higher levels of *Imbrasia belina* meal increased the levels of amylase, which was conferred to the remaining vegetable contents of *Imbrasia belina*, more than to the intrinsic components of that insect. Vegetables are the most common substrates for the feeding of insects. As part of the private knowledge of the insect provider, these data are not available in the present study, but the results in the amylase activity could be due, on the one hand, to different feeding habits of the insects, and on the other hand, to the different levels of wheat meal between H18 and T18 diets.

### 3.4. Liver Intermediary Metabolism

No differences were found between treatments for any of the measured enzymes (FBPase, PK, GPT, GOT and GDH), which means that intermediary metabolism in the liver was mostly unaffected (Table 5). As stated previously, protein use (Table 3) was in general more efficient on T18 than on H18, which gives the idea that protein availability was better for fish fed with T18. The literature concerning the analysis of intermediary liver metabolism after a feeding trial with IMs is scarce, but supports the point that these ingredients do not disrupt the function of these enzymes [23,28,94,95]. In a previous experience [32] with similar diets and rearing conditions, but with 10% inclusion level of IMs, an increase in GOT activity was observed; in the present study, even though T18 showed a very similar trend (*p*-value = 0.059), the ANOVA did not reveal a significant difference. Considering that liver histology did not show differences either, this also supports the present data.

### 3.5. Non-Specific Immune Status

The immune status was evaluated in different tissues. Tumour necrosis factor-alpha (TNF-α) was determined as a pro-inflammatory indicator [96] in distal intestine, as a first immune barrier from which an immune response can be initiated [97], and in skin mucus, for its role in innate immunity and fish health [98]. In addition, different parameters in plasma related to non-specific immune responses were determined.

No significant differences were found between treatments for TNF-α in distal intestine and skin mucus, or for lysozyme, esterase, anti-protease, alkaline phosphatase, peroxidase, or total immunoglobulins (IG) in plasma (Table 6).

The only statistically significant difference was on acid phosphatase in plasma, where T18 showed the lowest level, being different to C (*p* < 0.05; Table 6). Acid phosphatase is related to tissue damage in other species [99,100]. IMs are known for causing varied effects on the immunological system of fish, while the precise mechanisms that produce them are still unknown [14,30,33,88]. In general, the immunostimulant effect of Ims is well accepted [30,101,102], and a frequent justification for this is the influence of chitin and its derivatives, together with the presence of antimicrobial peptides in insects [13,103]. However, a publication by Xu [104] described how the replacement of soybean oil with an ω-3 enriched insect oil modified the genetic expression of IL-1β, IL-10, and TNF-α on the liver and kidney of juvenile mirror carp, as well as the amount of serum lysozyme. Moreover, Kumar [88] carried out two parallel experiments, one based on the addition of HI meal, and one on the replacement of fish oil with insect oil, and described different results for the same immunological parameters and the same organs of rainbow trout. Because the immunological system is complex and multifactorial, the different components of IMs such as chitin, insect fat, or other that might not be considered, could lead to very different interactions with it. Even though these immunological benefits are usually attributed to IMs, its chitin, or both [30,101], concluding results will not be reached until the precise mechanisms involved are described.

### 3.6. Liver Antioxidant Status and Fish Welfare Indicators

With the exception of GPx activity, which was higher for C than for IM-based diets (*p* < 0.05), T18 showed an overall more active antioxidant status. There were no differences for G6PDH or GR. SOD activity was higher in T18 than in C and H18 (*p* < 0.05). Higher levels of CAT activity were highlighted in T18 than in H18, and lower levels of MDA are described in T18 than in C diet (*p* < 0.05). No differences were found between treatments for glucose or lactate plasmatic levels (Table 7).

The antioxidant system is a complex biochemical structure composed of several molecules and enzymes that fight against the derived toxicity of reactive oxygen species (ROS) resulting from cellular metabolic processes. Roughly, SOD, CAT, and GPx are involved as direct defensive mechanisms against ROS, while GR regenerates the substrate of GPx (glutathione) and G6PDH works on the maintenance of this system by providing NADPH, which is used as a fuel to allow the activity of GR. An imbalance between ROS production and antioxidant mechanisms derivate in oxidative stress, resulting in cellular membrane lipids damage. MDA is a product formed from the breakdown of polyunsaturated fatty acids due to lipid peroxidation, and it may be used as a marker of cellular damage [105]. Because all these molecules work together to prevent oxidative injury, it is not strange to find coincidences in their activities, such as the ones shown between SOD and CAT, higher on T18, as well as its consequential MDA decrease on T18. This would also be in consonance with the previously mentioned results of plasmatic acid phosphatase, because a more efficient antioxidant system should be reflected on a lesser amount of cellular damage. In general, the current bibliography strongly supports the idea that IMs help to prevent the derived toxicity of oxygen in two different ways: indirectly, by enhancing the antioxidant system, or directly, by preventing the oxidative damage itself. The first case can be easily recognized in those experiences where higher activities of antioxidant elements are highlighted, ideally, but not always, followed by a consequential decrease in the concentration of oxidative damage indicators (typically, MDA) when a significant amount of IM is added to the diet [21,30,31,32]. The second case can be more complicated, because the mechanisms that regulate the reduction of oxidative damage through the addition of IMs are not yet well known. As previously stated for the immunological system, a frequent hypothesis used to justify this involves the activity of chitin and its derivates in fish physiology, because it was described that this molecule could produce a direct scavenging effect on radical species, as well as an increase in intracellular glutathione [29]. The work of Moutinho [20] described a decrease in both SOD and CAT activities and a lower concentration of MDA on European seabass liver after an increasing amount of HI was added to the diet, suggesting this preventive effect. Moreover, the work of Sánchez-Muros on tilapia [106] described an increase in liver SOD activity, muscle ROS, and intestine ferric-reducing antioxidant power for a diet based on FM and soy meal, while two experimental diets with TM meals produced the opposite effects, even though one of them had the same amount of soy meal as the first, giving again the idea of a preventive effect. Interestingly, the work of Xu [104] highlighted an increase in SOD activity in the liver of juvenile mirror carp after the administration of HI oil, which suggests that chitin might not be the only element involved in the enhancement of the antioxidant system of fish after using whole IMs.

Glucose and lactate are known to be indicators of animal welfare, because their levels in plasma are increased after stressful situations [107,108,109]. Other trials based on the evaluation of IMs as ingredients for fish compared the levels of plasmatic glucose, but in general, not many changes have been described [110,111]. The present study matched this case; on the one hand, this means that the fish homeostasis was correct and compensated even in those cases where other indicators (acid phosphatase and MDA) suggested the presence of tissue damage; on the other hand, IMs did not affect either of these animal welfare indicators in a positive way.

### 3.7. Proximate Composition of the Fillet

The proximate composition of rainbow trout fillets was appointed in Table 8. Moisture levels were lower for T18 than for C. Protein and ash levels were higher for insect-based treatments than for C diet, while fat showed no significant differences. Despite the lower values of phosphorus in IMs, the phosphorus content in the fillets was similar among treatments, so IMs were able to satisfy the nutritional requirements.

In general, dry components were higher on fillets of fish fed with insect-based diets. Several studies have evaluated the composition of rainbow trout fillets after a growth trial with HI and TM IMs. Some of them described no differences in fish fillet compositions [62,67,112], while others described small changes in raw protein or lipids, the decrease on these parameters being more frequent than the opposite trend [61,65,113]. However, all the cited manuscripts describe experiences with bigger sizes of rainbow trout than those used for the present study. Our previous study [32] did not show any differences on fillet protein, fat, or ash, which means that the increased inclusion of HI and TM in the diets (18% vs. 5/10%) may have played an important role on these changes. Considering this, it is possible that smaller fish could have dealt differently with higher amounts of IMs, contrary to what was described for bigger fish.

## 4. Conclusions

This study shows the importance of adequately selecting a type of IM before its inclusion as an ingredient in aquafeeds. Although in terms of absolute values for growth performance, the use of HI or TM in feeds for rainbow trout was efficient, fish fed with TM grew better than fish fed with HI. These differences have been marked by the higher use of the protein and more active digestive function, supported with intestinal histological changes observed, particularly the increase in villi height for T18. It is also remarkable that a small increase in enterocyte height was described for insect-based diets, which could be related to the different absorption of insect fat.

No changes were noticed for liver histology or intermediary metabolism. The antioxidant and immunological systems suffered a slight activity improvement for insect-based diets reflected on the decrease in tissue damage indicators (MDA and acid phosphatase), but this did not modify the overall health and welfare status of fish. Although more research is encouraged to isolate and identify the specific physiological mechanisms that make IMs improve the performance of both the antioxidant and the immunological systems of fish, this study supports the idea that IMs act as potential functional ingredients.

Minor changes in the composition of the fillets were observed, with a higher amount of protein in fish fed with insects. More research is encouraged to elucidate the long-term feeding effects.

## Figures and Tables

**Table 1:** Proximate and amino acids compositions of insect meals (IMs).

Proximate Composition	*Hermetia ilucens* (HI)	*Tenebrio molitor* (TM)
Crude protein (%)	28.5	39.1
Crude fat (%)	25.6	27.0
Crude ash (%)	9.75	3.42
Moisture (%)	8.00	5.00
Calcium (g/Kg)	35.2	0.93
Phosphorus (g/Kg)	7.00	7.50
Calcium–phosphorus ratio	5.03	0.12
Chitin (%)	7.50	5.90
**Amino acid composition (g/100 g IM)**		
Asp (aspartate)	2.92	3.71
Thr (threonine)	0.95	1.44
Ser (serine)	1.43	2.49
Glu (glutamate)	3.19	4.98
Pro (proline)	1.58	3.04
Gly (glycine)	1.84	2.87
Ala (alanine)	2.37	3.92
Cys (cysteine)	0.13	0.24
Val (valine)	1.42	2.32
Met (methionine)	0.47	0.57
Ile (isoleucine)	0.91	1.31
Leu (leucine)	1.86	2.96
Tyr (tyrosine)	2.23	4.47
Phe (phenylalanine)	2.16	3.07
His (histidine)	1.07	1.77
Lys (lysine)	1.94	2.49
Arg (arginine)	1.24	1.81

**Table 2:** Formulation, proximate, and amino acid composition of experimental diets.

Ingredients (%; on Wet Basis)	C	H18	T18
Fishmeal	35.9	18.0	18.0
HI meal	0.00	18.0	0.00
TM meal	0.00	0.00	18.0
Wheat gluten	10.5	15.4	11.9
Soy protein concentrate	15.5	18.3	17.0
Wheat meal	16.4	11.5	17.0
Soy lecithin	1.30	0.50	0.50
Fish oil	12.2	9.50	9.00
Vitamins and minerals	2.00	2.00	2.00
Goma guar	2.00	2.00	2.00
Blood meal	4.00	4.00	4.00
Methionine	0.20	0.50	0.50
Lysine	0.00	0.40	0.10
Total	100	100	100
**Proximate composition (%; on wet basis)**			
Moisture	7.43	7.74	7.21
Total crude protein	43.9	42.8	43.1
Total crude fat	17.2	17.1	17.9
Ash	7.41	6.45	6.11
Calcium	0.51	0.26	0.45
Phosphorus	0.33	0.24	0.24
Calcium–phosphorus ratio	1.6	1.1	1.9
Amino acid composition * (g/100 g feed)			
Asp (aspartate)	2.89	1.86	2.29
Thr (threonine)	1.65	1.25	1.38
Ser (serine)	1.59	1.22	1.46
Glu (glutamate)	6.55	5.96	5.96
Pro (proline)	2.15	2.11	1.84
Gly (glycine)	1.78	1.16	1.53
Ala (alanine)	1.73	1.05	1.58
Cys (cysteine)	0.68	0.67	0.64
Val (valine)	2.07	1.65	1.87
Met (methionine)	1.22	1.13	1.26
Ile (isoleucine)	1.93	1.55	1.63
Leu (leucine)	3.15	2.56	2.79
Tyr (tyrosine)	1.11	0.87	1.42
Phe (phenylalanine)	1.89	1.63	1.93
His (histidine)	1.03	0.84	1.03
Lys (lysine)	2.66	1.88	2.18
Arg (arginine)	2.50	1.86	2.04

Vitamin and mineral premix (% unless otherwise specified): vitamin A 2,000,000 UI; vitamin D3: 200,000 UI; vitamin E: 1.2; vitamin K3: 0.26; vitamin B1: 0.3; vitamin B2: 0.3; vitamin B6: 0.2; vitamin B9: 0.15; vitamin B12: 0.001; vitamin H: 0.03; inositol: 5; betaine: 5; calcium pantothenate: 1; nicotic acid: 2; Co: 0.006; Cu: 0.09; Fe: 0.06; I: 0.005; Mn: 0.095; Se: 0.0001; Zn: 0.075; Ca: 19; K: 2.4; Na: 4.1. C: control diet (no IM inclusion); H18: 18% *Hermetia illucens* inclusion (HI); T18: 18% *Tenebrio molitor* inclusion (TM). * Calculated from basic amino acids of ingredients.

**Table 3:** Growth performance, protein utilization, and biometric indices of rainbow trout fed experimental diets.

Growth Performance	C	H18	T18	SEM	*p*-Value
IBW (g)	14.3	14.8	14.7	0.22	0.325
IBL (cm)	11.1	11.2	11.2	0.05	0.377
FBW (g)	76.4 ^a,b^	69.4 ^b^	81.9 ^a^	2.41	0.016
FBL (cm)	18.0 ^a,b^	17.6 ^b^	18.5 ^a^	0.16	0.010
SGR (%/day)	2.17 ^a^	2.00 ^b^	2.23 ^a^	0.04	0.011
DFI (g/100 g fish·day)	1.57	1.62	1.57	0.02	0.267
FCR	0.90 ^a^	0.98 ^b^	0.88 ^a^	0.02	0.006
**Protein utilization**					
PER	2.49 ^a,b^	2.34 ^b^	2.58 ^a^	0.05	0.015
PPV (%)	49.2	48.6	51.8	1.23	0.196
ADC_prot_ (%)	92.6 ^a^	81.0 ^b^	91.2 ^a^	1.05	<0.0001
**Biometric indices**					
CF (g/cm^3^)	1.31	1.28	1.3	0.01	0.461
HSI (%)	1.26	1.44	1.29	0.07	0.244
VSI (%)	14.3	15.9	14.5	0.42	0.051

C: control diet (no IM inclusion); H18: 18% HI inclusion; T18: 18% TM inclusion. IBW: initial body weight; IBL: initial body length; FBW: final body weight; FBL: final body length; SGR (specific growth rate) = ((ln FBW-ln IBW)/days) · 100; DFI (daily feed intake) = (daily feed consumption (g)/biomass (g) at time) · 100; FCR (feed conversion ratio) = (total feed intake (g)/(FBW-IBW)); PER (protein efficiency ratio) = (total weight gain (g)/protein intake (g)); PPV (productive protein value) = ((protein gain (g)/protein intake (g)) · 100); ADC_prot_ (apparent digestibility coefficient of the protein) = 100 – ((marker in diet (g)/marker in faeces(g)) · (% protein in faeces/% protein in diet) · 100); CF (condition factor) = (weight (g)/length^3^ (cm)) · 100; HSI (hepatosomatic index) = (wet liver weight/FBW) · 100; VSI (viscerosomatic index) = (wet visceral weight/FBW) · 100. ^a, b^ Show statistically significant differences among diets (*p* < 0.05); Values are expressed as mean ± standard error of the mean (SEM; *n* = 4 tanks per diet).

**Table 4:** Digestive enzymes of rainbow trout fed experimental diets.

Digestive Enzymes (U/mg Protein)	C	H18	T18	SEM	*p*-Value
Acidic proteases	400.9	314.9	383.4	47.6	0.470
Alkaline proteases	82.2 ^b^	117.0 ^b^	263.5 ^a^	23.4	0.001
Acidic–alkaline ratio	6.36 ^a^	2.60 ^a,b^	1.49 ^b^	0.90	0.009
Amylase	81.5 ^b^	85.1 ^a,b^	139.5 ^a^	15.2	0.043

C: control diet (no IM inclusion); H18: 18% HI inclusion; T18: 18% TM inclusion. ^a, b^ Show statistically significant differences among diets (*p* < 0.05); Values are expressed as mean ± standard error of the mean (SEM; *n* = 4 tanks per diet, 2 fish per tank).

**Table 5:** Intermediary metabolism enzymes in liver of rainbow trout fed experimental diets.

Enzymes (mU/mg Protein)	C	H18	T18	SEM	*p*-Value
Fructose 1,6- biphosphatase (FBPase)	26.6	22.8	27.7	4.49	0.724
Pyruvate kinase (PK)	55.1	50.2	64.9	6.84	0.346
Glutamate pyruvate transaminase (GPT)	329.3	371.5	333.2	30.1	0.568
Glutamate oxaloacetate transaminase (GOT)	270.3	169.3	326.3	40.1	0.059
Glutamate dehydrogenase (GDH)	574.6	379.4	523.0	65.9	0.151

C: control diet (no IM inclusion); H18: 18% HI inclusion; T18: 18% TM inclusion. Values are expressed as mean ± standard error of the mean (SEM; *n* = 4 tanks per diet, 2 fish per tank).

**Table 6:** Non-specific immune parameters of rainbow trout fed experimental diets.

Immune Parameters	C	H18	T18	SEM	*p*-Value
TNF-α DI	0.43	0.36	0.59	0.08	0.189
TNF-α SM	0.75	0.37	0.52	0.11	0.105
Lysozyme	4.76	5.03	3.95	0.47	0.288
Esterase	1014.0	1114.9	1451.5	228.9	0.405
Acid phosphatase	882.1 ^a^	791.2 ^a,b^	581.9 ^b^	53.9	0.009
Alkaline phosphatase	2350.4	2215.5	1780.8	291.6	0.392
Anti-protease activity	91.8	101.2	103.7	7.56	0.525
Peroxidase	0.58	0.69	0.47	0.08	0.223
Total Immunoglobulins	12.2	14.5	16.3	1.51	0.205

C: control diet (no IM inclusion); H18: 18% HI inclusion; T18: 18% TM inclusion. TNF-α D.I.: Concentration of Tumour Necrosis Factor alpha in distal intestine; TNF-α S.M.: Concentration of Tumour Necrosis Factor alpha in skin mucus expressed as μg/mL; Lysozyme activity expressed as μg/mL HEWL (Hen Egg White Lysozyme); Esterase, acid and alkaline phosphatases as mU/mg protein; anti-protease activity as U anti-protease/mg protein; peroxidase as U/mg protein; total IG as mg/mL. ^a, b^ Show statistically significant differences among diets (*p* < 0.05). Values are expressed as mean ± standard error of the mean (SEM; *n* = 4 tanks per diet, 2 fish per tank).

**Table 7:** Liver antioxidant performance and fish welfare plasmatic parameters of rainbow trout fed experimental diets.

Antioxidant Enzymes and Lipid Peroxidation	C	H18	T18	SEM	*p*-Value
Superoxide Dismutase (SOD)	208.1 ^b^	209.7 ^b^	273.3 ^a^	15.3	0.023
Catalase (CAT)	186.5 ^a,b^	167.7 ^b^	216.7 ^a^	11.6	0.043
Glutathione Peroxidase (GPx)	12.2 ^a^	10.1 ^b^	9.85 ^b^	0.32	0.001
Glutathione Reductase (GR)	7.02	7.82	6.36	0.46	0.136
Glucose-6-phosphate dehydrogenase (G6PDH)	43.2	41.5	41.6	2.71	0.887
Malondialdehyde (MDA)	21.3 ^a^	15.7 ^a,b^	11.1 ^b^	2.40	0.043
**Fish welfare indicators**					
Glucose	3.33	3.69	3.23	0.19	0.319
Lactate	1.85	2.38	2.53	0.32	0.540

C: control diet (no IM inclusion); H18: 18% HI inclusion; T18: 18% TM inclusion. SOD and CAT expressed as U/mg protein; GPX, GR and G6PDH as mU/mg protein; MDA as nmol/g tissue; Glucose and lactate as mmol/L. ^a, b^ show statistically significant differences among diets (*p* < 0.05). Values are expressed as mean ± standard error of the mean (SEM; *n* = 4 tanks per diet, 2 fish per tank).

**Table 8:** Proximate composition of fillets of rainbow trout fed experimental diets.

Proximate Composition of the Fillet (Wet Basis)	C	H18	T18	SEM	*p*-Value
Moisture (%)	77.6 ^a^	77.2 ^a,b^	76.4 ^b^	0.24	0.017
Protein (%)	18.2 ^b^	19.6 ^a^	19.5 ^a^	0.24	0.006
Fat (%)	1.09	1.87	1.52	0.21	0.070
Ash (%)	1.29 ^b^	1.42 ^a^	1.38 ^a^	0.01	0.0001
Calcium (mg/Kg)	325.4	418.0	400.0	46.2	0.365
Phosphorus (g/Kg)	2.98	2.92	2.96	0.06	0.813

C: control diet (no IM inclusion); H18: 18% HI inclusion; T18: 18% TM inclusion. ^a, b^ Show statistically significant differences among diets (*p* < 0.05). Values are expressed as mean ± standard error of the mean (SEM; *n* = 4 tanks per diet, 2 fish per tank).

## Data Availability

The data presented in this study are available in the article.
